# Occurrence and Risk Assessment of Fluoroquinolone Residues in Chicken and Pork in China

**DOI:** 10.3390/antibiotics11101292

**Published:** 2022-09-22

**Authors:** Zhixin Fei, Shufeng Song, Xin Yang, Dingguo Jiang, Jie Gao, Dajin Yang

**Affiliations:** 1 NHC Key Laboratory of Food Safety Risk Assessment, China National Center for Food Safety Risk Assessment, Beijing 100022, China; 2 Yunnan Center for Disease Control and Prevention, Kunming 650022, China

**Keywords:** occurrence, risk assessment, fluoroquinolone, chicken, pork, China

## Abstract

Antibiotics, especially fluoroquinolones, have been exhaustively used in animal husbandry. However, very limited information on the occurrence and exposure assessment of fluoroquinolone residues in chicken and pork in China is available to date. Thus, a total of 1754 chicken samples and 1712 pork samples were collected from 25 provinces in China and tested by ultra-high performance liquid chromatography-tandem mass spectrometry (UPLC–MS/MS) for residual determination of six common fluoroquinolones. The results revealed that the detection frequencies of fluoroquinolone residues were 3.99% and 1.69% in chicken and pork samples. The overall violation frequencies were 0.68% and 0.41% for chicken and pork. Enrofloxacin and its metabolite ciprofloxacin were found to be the most predominant fluoroquinolones. The occurrence of these antibiotics in different sampling regions and market types was analyzed. The %ADI values of enrofloxacin and ciprofloxacin were far less than 100, indicating the health risk associated with the exposure to these aforementioned fluoroquinolone residues via chicken and pork for Chinese children, adolescents, and adults was acceptable. The results provided useful references for Chinese consumers, and helped to appropriately use these antibiotics in poultry and livestock industry.

## 1. Introduction

Chicken and pork are two of the most commonly consumed meats in China. Over the past 30 years, the per capita consumption of meat in China has increased by 50% [1]. The growth in demand has meant that the poultry and livestock industries have shifted from traditional family farming to intensive farming [2]. Nevertheless, as any intensive animal production system, the risk of the emergence and spread of infectious diseases are high [3]. Antibiotics have become an increasingly indispensable solution to protect food-producing animals from disease endangerments, either prophylactically or therapeutically, and avoid economic losses. Unfortunately, the use of huge amounts of antibiotics can result in the presence of their residues, and adverse effects on consumers and the environment. Antibiotic residues in the tissues of animals have raised several safety questions regarding allergenic potential, toxic effects (neurotoxicity, hepatotoxicity, nephrotoxicity, genotoxic effects and arthrotoxicity) and, more alarming, the development of antimicrobial resistance [4,5,6,7,8,9]. In addition, more than 70% of the antibiotics applied are then excreted into the environment via urine and feces [10]. The residual antibiotics in the environment may lead to potentially negative impacts on nontarget organisms, contamination of food and drinking water, and increase antibiotic resistance [11]. Because of long-term antibiotic use during animal breeding, antibiotic resistance has markedly increased in recent decades, which currently has become one of the most serious threats to human health [12,13,14].

Fluoroquinolones are a group of antibiotics exhaustively used in human and veterinary medicine, and act by inhibiting bacterial DNA gyrase and/or topoisomerase IV. After administration, fluoroquinolones exhibit rapid absorption with wide tissue dissemination and are excreted through urine and bile [15]. Usually, higher concentrations of drug residues are found in the liver and kidney, considering that the hepatobiliary system and the kidneys are the main routes by which drugs and their metabolites leave the body [16]. Owing to fluoroquinolones’ lipophilic characteristics, they possess a long half-life, and their metabolization is slow [17]. Fluoroquinolone residues can pose health hazards to consumers, and cause joint injury and allergic reactions, inducing unscheduled DNA synthesis, DNA strand breakage, and chromosome damage [15,18]. Furthermore, fluoroquinolone-resistance bacterial strains have been widely reported [6,19]. Many studies demonstrated that the resistant strains of *Campylobacter* spp., *Salmonella* spp., and *Escherichia coli* toward fluoroquinolones have been positively correlated with their use in animal production [20,21,22]. There is a high risk of transmitting these resistant strains to humans via the food chain, which makes infections difficult to treat [5].

Enrofloxacin is one of the most commonly used fluoroquinolone drugs in food-producing animals, and one of its major metabolites, ciprofloxacin is often found in animal tissue [4,23]. Although enrofloxacin has not been approved for use in poultry in the United States, it is extensively used in Latin America, Asia, and the European Union [4,6]. To standardize the use of enrofloxacin and ensure its residual concentration in animal-derived foods at an acceptable level, China has established a maximum residue level (MRL), which is calculated as the sum of enrofloxacin and ciprofloxacin. In poultry and pig, the defined MRLs are 100, 200, and 300 μg/kg for muscle, liver, and kidney tissues, respectively [24]. It is noteworthy that the withdrawal periods of 8 days for chicken and 10 days for pigs were enough to decrease the levels of enrofloxacin and ciprofloxacin below the permitted MRLs [25]. However, a longer time is needed from the last administration until residues are no longer detected. Moreover, for fluoroquinolone drugs without MRLs, a zero-tolerance principle applies. For example, norfloxacin, ofloxacin, pefloxacin, and lomefloxacin are used in human medicine, but are not allowed as veterinary medicine in food-producing animals in China [26]. 

In light of the above, monitoring and assessing dietary exposure risk to fluoroquinolone residues are essential to ensure the safety of the animal-based foods available to consumers. Several studies have reported fluoroquinolone residue levels in chicken or/and pork from other countries [4,27,28,29,30,31,32,33,34]. However, so far, limited information on the occurrence and exposure assessment of fluoroquinolone residues in chicken and pork in China is available. 

The primary goal of the present work was to investigate the occurrence and exposure risk of fluoroquinolone residues in chicken and pork in China. A national survey was conducted and 1754 chicken and 1712 pork samples were collected from 25 provinces across China. The presence and levels of six common fluoroquinolones were acquired using ultra-high performance liquid chromatography-tandem mass spectrometry (UPLC–MS/MS). Additionally, the residual levels acquired were further combined with food consumption data so as to estimate the exposure of fluoroquinolone residues to the consumers in China, and the potential health risks were conducted. To the best of our knowledge, this is the first comprehensive study on the occurrence and risk assessment of fluoroquinolones in chicken and pork in China.

## 2. Results and Discussion

### 2.1. Occurrence of Fluoroquinolone Residues in Chicken and Pork

The occurrence and residue levels of six fluoroquinolones in chicken and pork are summarized in Table 1. Overall, the detection frequencies of these antibiotics were 3.99% and 1.69% in chicken and pork samples, respectively. The overall violation frequencies of exceeding MRLs and misusing banned antibiotics in samples were 0.68% and 0.41% for chicken and pork. It can be easily seen that the occurrence and levels of fluoroquinolone residues in chicken were higher than those in pork in China. 

It was found that the detection frequency of enrofloxacin was the highest among all the individual fluoroquinolones, followed by ciprofloxacin, in both pork and chicken (Table 1). In chicken, enrofloxacin occurred with a detection frequency of 3.36%, a mean concentration of 2.07 μg/kg, and a maximum concentration of 1280 μg/kg. In contrast, ciprofloxacin occurred with a lower detection frequency of 1.14%, a mean concentration of 0.20 μg/kg, and a maximum concentration of 45.3 μg/kg. A sum of enrofloxacin and ciprofloxacin residue was detected in 3.82% of chicken samples. In pork, enrofloxacin was detected in 25 samples (1.46%) with a mean concentration of 0.74 μg/kg and a maximum concentration of 529 μg/kg, and ciprofloxacin was found in 13 samples (0.76%), with a mean concentration of 0.14 μg/kg and a maximum concentration of 89.9 μg/kg. Enrofloxacin and/or ciprofloxacin were detected in 1.52% of pork samples. Moreover, 9 chicken samples (0.51%) and 4 pork samples (0.23%) exceeded the MRL of 100 μg/kg for the sum of enrofloxacin and ciprofloxacin (Table 1), which might result from inadequate withdrawal periods before slaughter, and/or inappropriate dosage [35].

**Figure 1:**
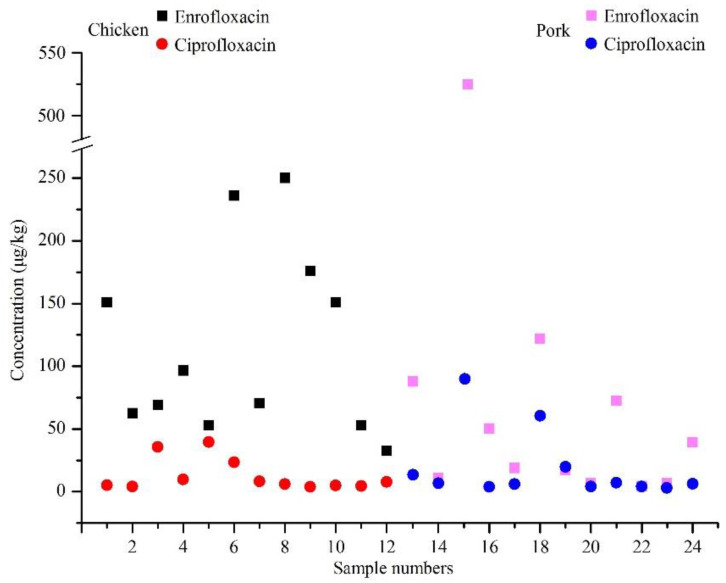
Concentrations of enrofloxacin and ciprofloxacin in the chicken and pork samples with the two fluoroquinolones detected simultaneously.

Because ciprofloxacin is a primary metabolite of enrofloxacin, the amount of ciprofloxacin increases according to the dose and duration of enrofloxacin administration. In this study, enrofloxacin and ciprofloxacin were simultaneously detected in 12 chicken and 12 pork samples, and concentrations of the two fluoroquinolones were compared in Figure 1. Almost all of the concentrations detected of enrofloxacin were high than ciprofloxacin except for one sample that the detection values of enrofloxacin and ciprofloxacin were 17.1 and 19.7 μg/kg, respectively. This result was in accordance with those of studies on pharmacokinetic in poultry and pigs, in which the concentrations of ciprofloxacin were lower than those of the parent drug enrofloxacin in the muscle after treated with enrofloxacin [36,37,38,39]. 

Meanwhile, we also detected prohibited fluoroquinolones in samples (Table 1). Ofloxacin was detected in one chicken sample with a concentration of 92.6 μg/kg, and in five pork samples with a maximum concentration of 848 μg/kg, respectively. Lomefloxacin was present in two chicken samples with a maximum concentration of 10.8 μg/kg. It is noteworthy that those prohibited fluoroquinolones were also detected in livestock and poultry products from some provinces of China in recent years, such as Shanghai [23], Fujian [40], and Xinjiang [41]. These results demonstrated that the illegal use of antibiotics still existed. 

### 2.2. Occurrence of Fluoroquinolone Residues in Different Regions

**Figure 2:**
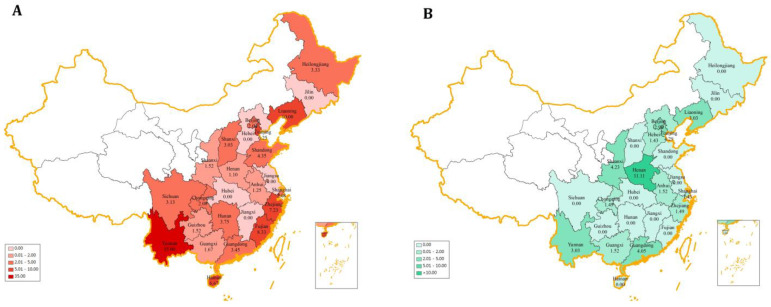
Occurrence of fluoroquinolone residues in different provinces (%). (**A**) chicken; (**B**) pork.

The regional distribution of fluoroquinolone residues in chicken and pork can be observed in Figure 1. The red (Figure 2A) and green (Figure 2B) coloring illustrate the detection frequencies of fluoroquinolone residues in chicken and pork, respectively, with darker colors representing higher detection frequency. In addition, the blanks indicate missing data. In chicken, Yunnan (35.00%) presented the highest detection frequency of fluoroquinolone, followed by Liaoning (10.00%), Fujian (8.33%), and Zhejiang (7.23%). The occurrence of fluoroquinolones in pork was lower than that in chicken with the exception of Anhui, Beijing, Guangdong, Henan, and Shaanxi. The provinces with higher detection frequencies in pork were mainly Henan (11.11%), Tianjing (4.29%), Shaanxi (4.23%), and Guangdong (4.05%). One should note that no antibiotics were detected in pork and chicken in Hubei, Jilin, Jiangsu, and Jiangxi. This study indicated that the occurrence of fluoroquinolone residues in chicken and pork varied considerably among different regions. Chicken in Yunnan and pork in Henan should be given more attention. It is necessary to strengthen the monitoring by expanding sample size in key provinces.

### 2.3. Occurrence of Fluoroquinolone Residues in Different Sampling Site Types

Table 2 shows the difference in fluoroquinolone residue occurrence between sampling site types. Regarding the samples from country fairs, fluoroquinolones were detected in 4.47% and 1.88% of the chicken and pork, respectively, and violation frequencies were 0.98% and 0.22%. Concerning samples from stores, fluoroquinolones were found in 3.46% and 1.48% of chicken and pork, respectively, and violation frequencies were 0.36% and 0.62%. Although there were higher detection frequencies in samples from country fairs, the results of statistical analysis showed that there was no significant difference in fluoroquinolone contaminations of chicken and pork between country fairs and stores (*p* > 0.05).

### 2.4. Comparison with Other Studies

The findings of this study were further compared to some of the data presented in other studies regarding the measurement of quinolones or fluoroquinolones in chicken meat. In a previous study, a total of 127 chicken meat samples were studied to detect quinolones from Ankara, Turkey, where 45.7% of samples were positive for quinolones and the mean level of quinolones was found to be 30.81 μg/kg [42]. In other studies, data on the occurrence of enrofloxacin or/and ciprofloxacin in chicken were found for Portugal [4,27], Indonesia [28], Korea [29], Lebanon [30], Sri Lanka [31], South Africa [32], and Vietnam [33], with a detected frequency in the range of 4.2–51.9% and 5.17–67.3%, respectively, which was higher than that observed in our study (3.36% and 1.14%). This result indicated that these antibiotics are widely used in the world. Moreover, there is a huge difference in the enrofloxacin and ciprofloxacin residues among different countries. For example, enrofloxacin was detected in Sri Lanka at a higher frequency of 51.9% compared to ciprofloxacin (7.0%) [31]. Similarly, seven (12.1%) of the chicken meat samples were positive for enrofloxacin, but only three (5.2%) of the chicken meat samples were positive for ciprofloxacin in Korea [29]. On the contrary, the detection frequencies of enrofloxacin residues were found to be lower than that of ciprofloxacin in chicken samples in Indonesia (41.8% and 67.3%), Lebanon (12.5% and 32.5%), and Portugal (51.0% and 60.4%) [4,28,30].

To our knowledge, there are only two reports on the residues of enrofloxacin and ciprofloxacin in pork. Ciprofloxacin residues were detected at mean concentrations of 315.30 μg/kg in 28 out of 80 pork samples collected from open markets in Ibadan, Nigeria [34]. Another study showed that enrofloxacin and ciprofloxacin were not detected in 19 pork samples in Shanghai, China [23].

Norfloxacin was found in 11.1% of the chicken samples from school canteens in Portugal, whereas it was not found in samples from supermarkets from 2013–2015 [4]. However, also in Portugal, 16% of the supermarket samples showed contamination with norfloxacin in 2010 [27]. The highest detection frequency for norfloxacin was observed in Nigeria, with 55% and 30% in chicken and pork, respectively [34]. Another study showed that the detection frequency for norfloxacin in chicken in Lebanon was 5%; furthermore, ofloxacin and lomefloxacin were detected at a frequency of 18.75% and 7.5% [30]. These studies reported a higher occurrence of the prohibited fluoroquinolones than that in our study. 

Although there were lower occurrences and levels of fluoroquinolone residue frequencies in chicken and pork meat in this study, the high frequencies of fluoroquinolone contaminations were found in other meat in China, such as beef, mutton, and fish. Zhang et al. analyzed 22 cattle muscle and 24 sheep muscle samples obtained from southern Xinjiang of China and found fluoroquinolone residue rates up to 63.64% and 62.50% [41]. Wang et al. reported detection frequencies of fluoroquinolones as 58.5% in fish from a total of 53 samples in Shanghai, China [23]. In addition, high detection frequencies for some prohibited fluoroquinolones were also observed in those studies, such as norfloxacin (18.18% in cattle muscle and 29.17% in sheep muscle) and ofloxacin (15.1% in fish). These results suggest that a national survey of fluoroquinolone residues in other animal-derived foods should be conducted in the future.

### 2.5. Risk Assessment

In general, risk assessment is the systematic characterization of potential adverse effects caused by exposure to hazardous agents. Dietary exposure assessment study is an important step for risk assessment procedure [43]. In our work, dietary exposure assessment of fluoroquinolones was performed using the residue levels of the fluoroquinolones in meat and food consumption of target specific groups of the population, including children, adolescents and adults. As norfloxacin and pefloxacin were not detected in chicken or pork, this study only estimated the dietary exposure to the other four fluoroquinolones. 

The results are summarized In Table 3. Regarding chicken and pork, the average EDIs of the four individual fluoroquinolones ranged from 0.003 to 0.965 ng/kg bw/day and from 0.155 to 1.328 ng/kg bw/day in all population groups, respectively. Although the residue levels of enrofloxacin and ciprofloxacin were higher in chicken than those in pork, and the exposure values of the two antibiotics in chicken were lower, owing to higher consumption of pork. The average EDIs for the sum of consumption of chicken and pork ranged from 0.003 to 2.633 ng/kg bw/day, while the EDIs in the worst-case scenario ranged from 2.8 to 1707.0 ng/kg bw/day, which was an extremely conservative estimation. In addition, we could clearly observe that the EDI values of each antibiotic in different age groups of the population followed the order of children > adolescents > adults, and all EDIs for children were ~70% higher than those for adults. This indicated that young consumers were more susceptible to various residues than adults [4,44]. Therefore, systematic exposure of antibiotics even in low concentrations, especially in early life, may have a negative impact in human health [43]. 

Due to the lack of health guidance value, it was not possible to undertake risk characterization of ofloxacin and lomefloxacin. The acceptable daily intake (ADI) of enrofloxacin (6.2 μg/kg bw/day) set by China [24] was used for risk characterization of enrofloxacin and its metabolite ciprofloxacin. Considering the consumption of chicken and pork, the %ADI values in the average scenario were 4.25 × 10^−2^, 2.86 × 10^−2^, and 2.52 × 10^−2^ for children, adolescents, and adults, respectively, which indicated a low health risk. Using the worst-case scenario approach, the consumption of chicken and pork accounted for 16.41–27.53% of the ADI, suggesting that the exposure risk is still acceptable for different age groups of the Chinese population. 

Nevertheless, in the present study, other food items that might contain fluoroquinolones, such as beef, fish, lamb, and eggs, were not considered. Further evaluation of dietary exposure to fluoroquinolones should be conducted. Furthermore, those drug residues in food may lead to the development of bacterial resistance to human antibiotics, even if the contaminant concentration is low [3]. Consequently, continuous monitoring and risk assessment for fluoroquinolones in animal food is still greatly needed.

## 3. Materials and Methods

### 3.1. Sample Collection and Preparation

A total of 1754 raw chicken samples and 1712 raw pork samples were randomly collected from stores and country fairs located in 25 provinces (Anhui, Beijing, Fujian, Guangdong, Guangxi, Guizhou, Hainan, Hebei, Henan, Heilongjiang, Hubei, Hunan, Jilin, Jiangsu, Jiangxi, Liaoning, Shandong, Shanxi, Shaanxi, Shanghai, Sichuan, Tianjin, Yunnan, Zhejiang, and Chongqing) across China in 2019. These samples were later subjected to grinding in a laboratory blender and stored at −18 °C until the extraction procedure. 

### 3.2. Chemicals and Reagents

The standards of six fluoroquinolones, enrofloxacin, ciprofloxacin, ofloxacin, norfloxacin, pefloxacin, and lomefloxacin were of high purity grade (>95%) and purchased from Sigma-Aldrich (St. Louis, MO, USA) and Dr. Ehrenstorfer (Augsburg, Germany). Methanol and acetonitrile were of HPLC grade and purchased from Thermo Fisher (Thermo Fisher Scientific, Waltham, MA, USA) and J. T. Baker (Phillipsburg, NJ, USA). Formic acid was of HPLC grade and citrate, sodium hydrogen phosphate, and disodium ethylenediaminetetraacetate dihydrate (Na_2_EDTA) were all analytical grade. Ultra-pure water was prepared using a Milli-Q system (Bedford, MA, USA).

### 3.3. Extraction Procedures

All samples from different regions were analyzed using a confirmatory UPLC–MS/MS method as described by Shao et al. in local laboratories with some minor modifications [45]. Briefly, 2.0 g of the samples were separately weighed into a 50 mL polypropylene centrifuge tube with a screw cap. Subsequently, 20 mL of EDTA-McIlvaine buffer (0.1 mol/L) was added to the tube, followed by vortex mixing for 1 min. The sample was ultrasonically extracted for 10 min at room temperature, and centrifuged at 10,000 rpm for 5 min. Afterward, the supernatant was subjected to solid-phase extraction on an OASIS HLB cartridge (200 mg, 6 mL; Waters, Milford, MA, USA). The cartridge was sequentially preconditioned with 6 mL of methanol and 6 mL of ultrapure water. Then, the extract was applied to the cartridge at a flow rate of 2–3 mL/min and washed with 2 mL of a mixture of methanol/water 5/95 (*v*/*v*). The analytes were eluted with 6 mL of methanol into a new centrifuge tube. The eluate was evaporated to dryness under a flow of nitrogen, and 1 mL of 0.1% formic acid was added. The reconstituted solution was filtered through 0.22 μm filters for analysis.

### 3.4. Instrumental Analysis

Analysis was performed by UPLC–MS/MS system using an ACQUITY UPLC BEH C18 column (100 mm × 2.1 mm, 1.7 μm, Waters, Dublin, Ireland) at a flow rate of 0.2 mL/min; the column temperature was kept at 40 °C. The mobile phases consisted of 40% (*v*/*v*) methanol/acetonitrile (A) and 0.2% (*v*/*v*) formic acid solution (B). A gradient elution program was used: It started with 10% A; increased linearly to 30% A from 0 to 6.0 min; increased linearly to 50% A from 6.0 to 9.0 min; and increased linearly to 100% A from 9.0 to 9.5 min; kept at 100% A for 1.0 min, returned to the initial conditions at 11 min. The run time was 15 min for each injection. 

MS/MS acquisition was performed using electrospray ionization (ESI) in positive ion mode, and multiple reaction monitoring (MRM) mode was used to quantitatively determine. The source temperature and desolvation temperature were 110 and 350 °C, respectively. The capillary voltage was 2.0 kV. Mass parameters of six fluoroquinolones are shown in Table 4.

### 3.5. Quality Control and Quality Assurance

For each batch of 10~15 samples, one blank control and one matrix-spiked sample were analyzed. The mean recovery rates for all target analytes in the sample spiked were in the range of 75–125% with a relative standard deviation (RSD) of <20%. Linearity was confirmed on the basis of correlation coefficients R^2^ > 0.990 for all analytes. The limits of detection and quantitation (LOQ) were regarded as the concentrations that produced a signal-to-noise (S/N) ratio of 3 and 10, respectively, which were estimated from the matrix-spiked sample with the lowest fortification level for the individual analyte. The LODs and LOQs of the six fluoroquinolones were 3 and 10 μg/kg, respectively.

### 3.6. Statistical Analysis

All statistical analysis was performed using R statistical software (Version 4.1.1, R Core Team). The chi-square test and *t*-test were applied to test for differences. Results with a *p*-value of <0.05 were considered significant.

### 3.7. Risk Assessment

To obtain comprehensive information about consumer exposure, the estimated daily intake (EDI) of antibiotics for children, adolescents, and adults was calculated according to the following Equation (1) [3].

(1) $$\mathrm{EDI}=\frac{C\times IR}{BW\times 1000}$$

where C (μg/kg) is the content of the target fluoroquinolones in the chicken/pork samples. The mean and maximum concentrations of antibiotics were applied to set the average and the worst-case scenario [46], respectively. IR represents the daily consumption of meat for the population. According to the monitoring report on the nutrition and health status of Chinese residents from 2010 to 2013, the mean daily consumption of poultry/pork was 15.6/66.4, 17.3/64.1, and 13.8/53.1 g/day for an adult, 14–17 years for adolescents, and 7–10 years for children [47], respectively, which was used in this study. Finally, the term BW refers to the average body weight, which was 60 kg for adults, 53.9 kg for adolescents, and 29.6 kg for children [48,49].

The resulting dietary exposure estimate was then compared with the recommended ADI value obtained from toxicological assessments, as shown below the Equation (2):

(2) $$\%ADI=\frac{EDI}{ADI}\times 100$$

when %ADI < 100, the risk is acceptable or low risk; otherwise, %ADI > 100 indicates an unacceptable risk [43,48].

## 4. Conclusions

In this study, the occurrence and exposure risk of fluoroquinolone residues in chicken and pork in China was investigated. On the whole, the levels of fluoroquinolone residues in chicken were higher than those in pork, with detection frequencies of 3.99% and 1.69%, respectively. It is clear that the detection frequencies and mean concentrations were found to be highest for enrofloxacin, followed by ciprofloxacin, both in chicken and pork. Moreover, we detected prohibited fluoroquinolones (ofloxacin and lomefloxacin) in samples. The violation frequencies of fluoroquinolones in chicken and pork were found to be 0.68% and 0.41%, respectively. Due to higher consumption of pork, the EDI of enrofloxacin and ciprofloxacin from pork was higher than that from chicken. All EDI values of enrofloxacin and ciprofloxacin (0.588 to 1707.0 ng/kg bw/day) were lower than the ADI. Although the results of the dietary risk assessment indicated an acceptable risk for enrofloxacin and ciprofloxacin from chicken and pork in the different age groups of China population, continuous residue monitoring and risk evaluation of fluoroquinolones in animal food should be increased.

## Figures and Tables

**Table 1:** Occurrence and residue levels of the selected antibiotics and their MRLs.

Antibiotic		Chicken (*n* = 1754)					Pork (*n* = 1712)			
	DF (n, %)	Mean (μg/kg)	Min (μg/kg)	Max (μg/kg)	VF (m, %)	DF (n, %)	Mean (μg/kg)	Min (μg/kg)	Max (μg/kg)	VF (m, %)
Enrofloxacin	59, 3.36	2.07	3.05	1280	-	25, 1.46	0.74	4.81	529	-
Ciprofloxacin	20, 1.14	0.20	3.88	45.3	-	13, 0.76	0.14	3.88	89.9	-
Ciprofloxacin + Enrofloxacin	67, 3.82	2.26	3.05	1280	9, 0.51	26, 1.52	0.88	4.81	618.9	4, 0.23
Ofloxacin	1, 0.06	0.05	92.6	92.6	1, 0.06	5, 0.29	0.65	3.48	848	5, 0.29
Norfloxacin	0, 0.00	<LOD	<LOD	<LOD	0, 0.00	0, 0.00	<LOD	<LOD	<LOD	0, 0.00
Pefloxacin	0, 0.00	<LOD	<LOD	<LOD	0, 0.00	0, 0.00	<LOD	<LOD	<LOD	0, 0.00
Lomefloxacin	2, 0.11	0.01	10.5	10.8	2, 0.11	0, 0.00	<LOD	<LOD	<LOD	0, 0.00
*Fluoroquinolones*	70, 3.99	-	-	-	12, 0.68	29, 1.69	-	-	-	7, 0.41

Abbreviations: DF, detection frequency; VF, violation frequency; Mean, mean concentration; Min, minimum concentration; Max, maximum concentration; ND, non-detectable, NA, not available; not calculated; LOD, limits of detection.

**Table 2:** Occurrence of fluoroquinolone residues in country fairs and stores.

Sampling Site Types	Chicken		Pork	
	DF ^a^	VF ^b^	DF ^c^	VF ^d^
Country fairs	4.47% (41/917)	0.98% (9/917)	1.88% (17/903)	0.22% (2/903)
Stores	3.46% (29/837)	0.36% (3/837)	1.48% (12/809)	0.62% (5/809)

Abbreviations: DF, detection frequency; VF, violation frequency; ^a^ Within a column, there was no significant difference (χ^2^ = 1.157, *p* = 0.282, *p* > 0.05). ^b^ Within a column, there was no significant difference (χ^2^ = 2.500, *p* = 0.114, *p* > 0.05). ^c^ Within a column, there was no significant difference (χ^2^ = 0.409, *p* = 0.523, *p* > 0.05). ^d^ Within a column, there was no significant difference (χ^2^ = 0.818, *p* = 0.366, *p* > 0.05).

**Table 3:** Estimated daily exposure and risk assessment of fluoroquinolones in chicken and pork.

Sample	Antibiotic	Average Scenario Approach						Worst-Case Scenario Approach					
		Children		Adolescents		Adult		Children		Adolescents		Adult	
		EDI (ng/kg bw/day)	% ADI (×10^−2^)	EDI (ng/kg bw/day)	% ADI (×10^−2^)	EDI (ng/kg bw/day)	% ADI (×10^−2^)	EDI (ng/kg bw/day)	% ADI	EDI (ng/kg bw/day)	% ADI	EDI (ng/kg bw/day)	% ADI
Chicken	Ciprofloxacin	0.965	-	0.664	-	0.538	-	596.8	-	410.8	-	332.8	-
	Enrofloxacin	0.093	-	0.064	-	0.052	-	21.1	-	14.5	-	11.8	-
	Ciprofloxacin + Enrofloxacin	1.054	1.70	0.725	1.17	0.588	0.95	596.8	9.62	410.8	6.62	332.8	5.37
	Lomefloxacin	0.005	-	0.003	-	0.003	-	5.0	-	3.5	-	2.8	-
	Ofloxacin	0.023	-	0.016	-	0.013	-	43.2	-	29.7	-	24.1	-
Pork	Ciprofloxacin	1.328	-	0.880	-	0.819	-	949.0	-	629.1	-	585.4	-
	Enrofloxacin	0.251	-	0.166	-	0.155	-	161.3	-	106.9	-	99.5	-
	Ciprofloxacin + Enrofloxacin	1.579	2.55	1.046	1.69	0.974	1.57	1110.2	17.91	736	11.87	684.9	11.05
	Lomefloxacin	ND	-	ND	-	ND	-	ND	-	ND	-	ND	-
	Ofloxacin	1.166	-	0.773	-	0.719	-	1521.2	-	1008.5	-	938.4	-
Chicken + Pork	Ciprofloxacin	2.293	-	1.544	-	1.357	-	1545.8	-	1039.9	-	918.2	-
	Enrofloxacin	0.344	-	0.230	-	0.207	-	182.4	-	121.4	-	111.3	-
	Ciprofloxacin + Enrofloxacin	2.633	4.25	1.771	2.86	1.562	2.52	1707.0	27.53	1146.8	18.50	1017.7	16.41
	Lomefloxacin	0.005	-	0.003	-	0.003	-	5.0	-	3.5	-	2.8	-
	Ofloxacin	1.189	-	0.789	-	0.732	-	1564.4	-	1038.2	-	962.5	-

**Table 4:** UPLC–MS/MS parameters for six fluoroquinolones.

Antibiotic	Formula	Parention (m/z)	Daughter Ion (m/z)	Cone Voltage (V)	Collision Energy (eV)
Ciprofloxacin	C_17_H_18_N_3_FO_3_	332.2	314.3 */288.3	36/36	19/17
Enrofloxacin	C_19_H_22_FN_3_O_3_	360.3	316.4 */342.3	38/38	19/23
Lomefloxacin	C_17_H_19_F_2_N_3_O_3_	352.3	265.2 */308.3	36/36	23/17
Norfloxacin	C_16_H_18_FN_3_O_3_	320.3	302.3 */276.3	50/50	19/17
Ofloxacin	C_18_H_20_FN_3_O_4_	362.2	318.3 */261.2	38/38	18/27
Pefloxacin	C_17_H_20_FN_3_O_3_	334.3	290.3 */233.2	38/38	17/25

* Quantitative ion.

## Data Availability

All of the data supporting this article are included in the main text.
